# Supplementary material for: Mechanism of chaperone recruitment and retention on mitochondrial precursors
Source: Mol Biol Cell. Author manuscript; Available in PMC 2025 Apr 2. (PMC7617541; doi:10.1091/mbc.E25-01-0035)
Supplement: Comb Supp Mats [file EMS204165-supplement-Comb_Supp_Mats.pdf]

Supplemental Materials

Molecular Biology of the Cell

Juszkiewicz *et al*.

A

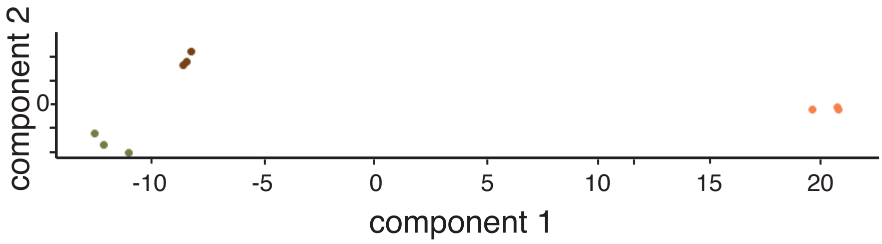

B

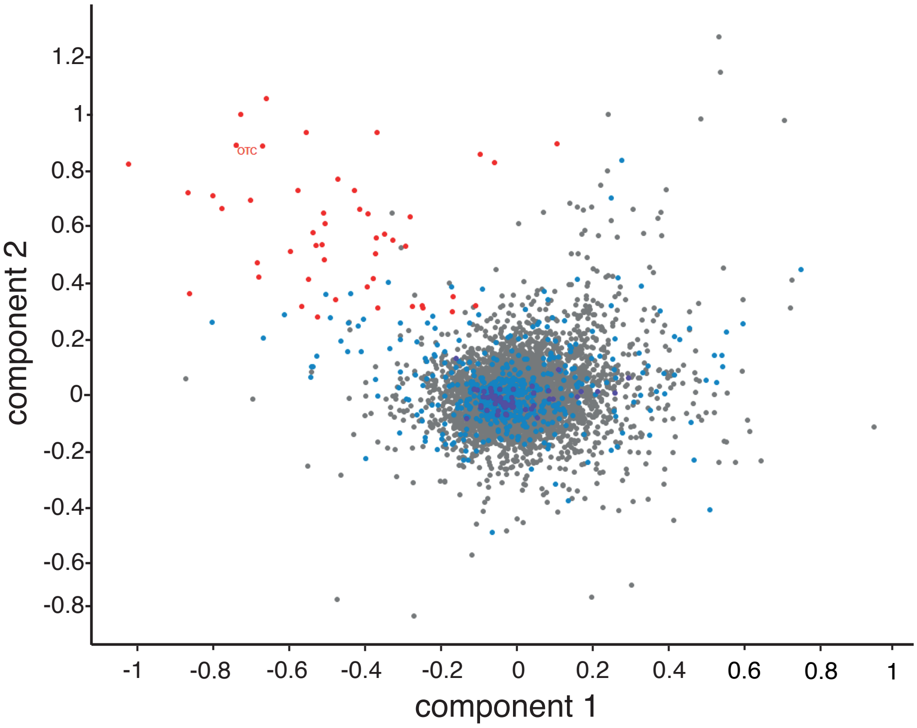

Figure S1. Hsp90 broadly buffers mitochondrial precursor degradation during import stress
(A) Principal component analysis (PCA) of the three technical replicates for each of the three biological conditions (depicted in three different colors) analyzed in the mass spectrometry experiment presented in Figure 2. Note clustering of the replicates indicative of high reproducibility of the results. **(B)** Principal component analysis (PCA) of the dataset from the the TMT experiment depicted in Figure 2C. Note that Hsp90 dependent proteins (red dots) tend to cluster together in the upper left quadrant of the plot.

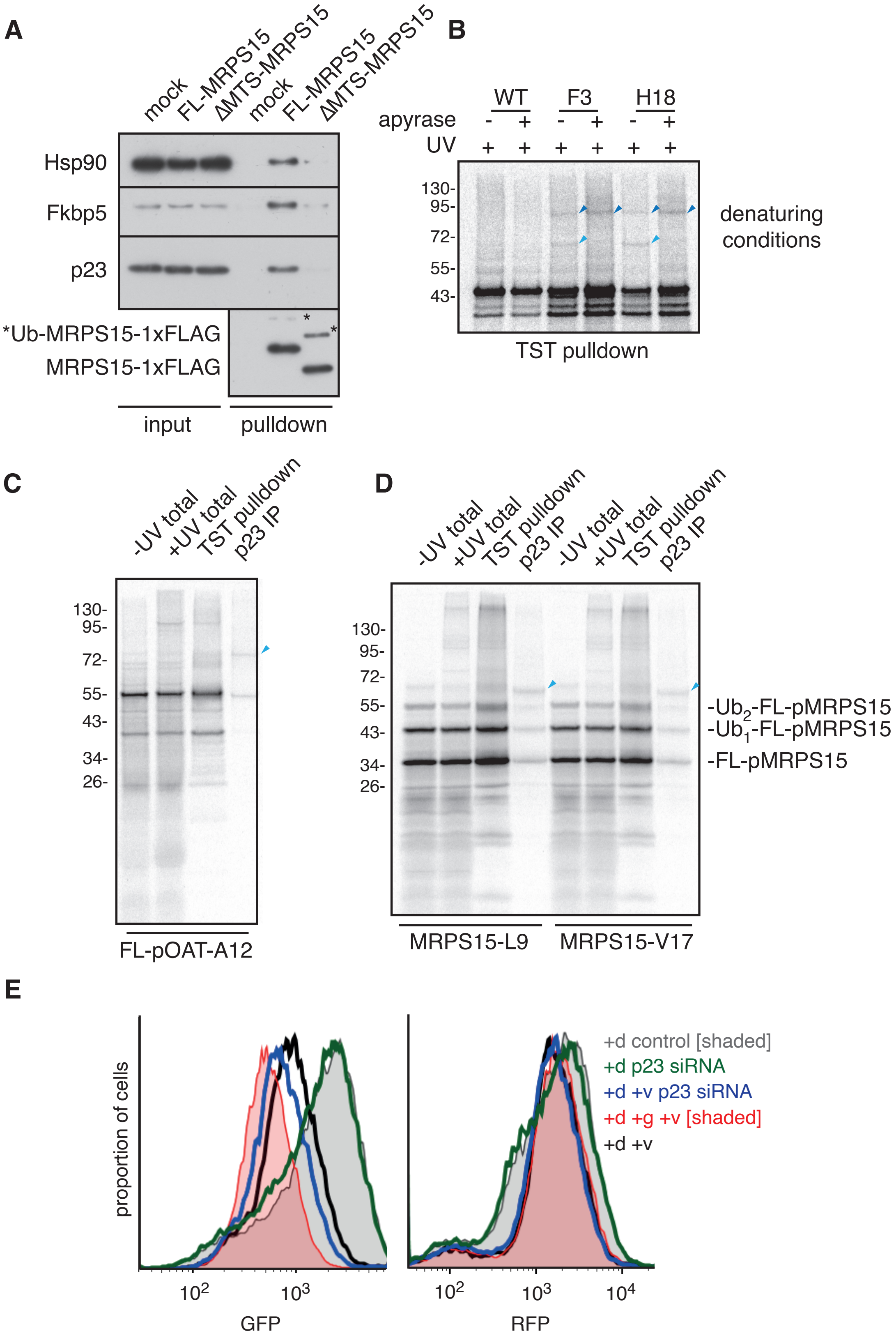
A
mock
FL-MRPS15
ΔMTS-MRPS15
mock
FL-MRPS15
ΔMTS-MRPS15
Hsp90
Fkbp5
p23
*Ub-MRPS15-1xFLAG
MRPS15-1xFLAG
input
pulldown
B
WT
F3
H18
apyrase
- + - + - +
UV
+ + + + + +
130-
95-
72-
55-
43-
denaturing conditions
TST pulldown
C
-UV total
+UV total
TST pulldown
p23 IP
130-
95-
72-
55-
43-
34-
26-
FL-pOAT-A12
D
-UV total
+UV total
TST pulldown
p23 IP
-UV total
+UV total
TST pulldown
p23 IP
130-
95-
72-
55-
43-
34-
26-
-Ub2-FL-pMRPS15
-Ub1-FL-pMRPS15
-FL-pMRPS15
MRPS15-L9
MRPS15-V17
E
proportion of cells
10^2
10^3
GFP
10^2
10^3
10^4
RFP
+d control [shaded]
+d p23 siRNA
+d +v p23 siRNA
+d +g +v [shaded]
+d +v

Figure S2. MTS facilitates Hsp90 retention.

(A) FL or ΔMTS MRPS15 was translated in RRL in the presence or absence of 2 μM geldanamycin and affinity purified under native conditions via a C-terminal FLAG tag. Samples eluted with FLAG peptide were analyzed by immunoblotting alongside input samples. Ubiquitinated MRPS15 is marked with asterisk. **(B)** FL-pOTC-TST with BpA crosslinker at positions F3 or H18 was produced in RRL. Samples were then UV irradiated either prior to or after ATP depletion with apyrase. All samples were affinity purified via the TST tag on the substrate and analyzed by SDS-PAGE and autoradography. Note the disappearence of the smaller crosslink (light blue arrow) upon ATP depletion. **(C)** FL-pOAT-TST with BpA crosslinker in the MTS (position A12) was produced in RRL and UV irradiated. Samples were then fully denatured and affinity purified using Streptactin (TST pulldown) or anti-p23 antibody. **(D)** MRPS15-TST with BpA at positions L9 or V17 was produced in RRL and UV irradiated. Samples were then fully denatured and affinity purified using Streptactin (TST pulldown) or anti-p23 antibody. **(E)** Histograms representing GFP or RFP signal from the experiment presented in Figure 3F. Note the selective decrease in GFP signal (without an appreciable change in RFP signal) in cells treated with siRNA against p23 (blue traces).

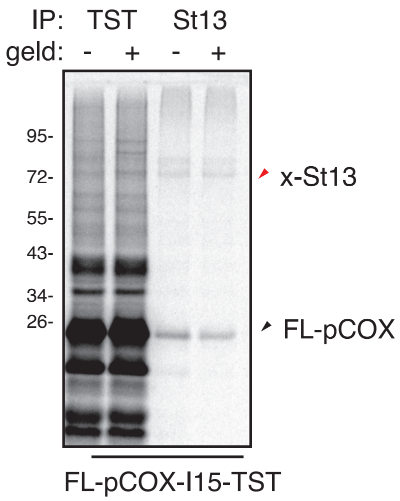

Figure S3. **St13 interact with the MTS of pCOXIVI1.**
BpA-containing pCOXIVI1 was produced in RRL in the presence or absence of geldanamycin and crosslinked with UV. Samples were then fully denatured and affinity purified using Streptactin (TST pulldown) or anti-St13 antibody. pCOX-St13 crosslink is marked with red arrow.

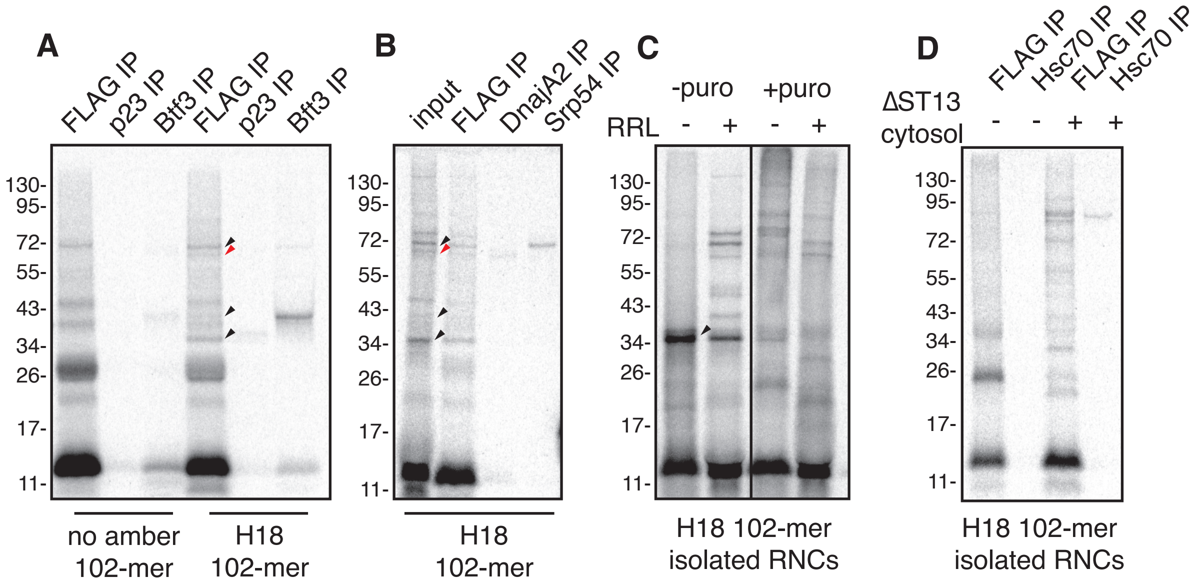

Figure S4. **St13 engages the MTS co-translationally and is retained after ribosome release.**
(A) 102-mer pOTC ribosome-nascent chain complexes (RNCs; see Figure 5A) with or without BpA at position H18 were produced in RRL. After UV crosslinking, samples were denatured and immunoprecipitated (IP) with antibodies against FLAG (substrate), p23 or Btf3. **(B)** An experiment as in panel A, but with IP using antibodies against DnajaA2 and Srp54. **(C)** 102-mer pOTC RNCs with BpA at positon H18 were synthesized in RRL and separated from bulk cytosol under high-salt conditions via centrifugation. The resuspended RNCs were mixed with RRL or buffer where indicated, released from ribosomes using puromycin where indicated, and subjected to UV irradiation. Note that of the several crosslinks observed in panel A, only the smallest of them is retained after RNCs are isolated in high salt (arrowhead). The lost crosslinks are restored if the RNCs are mixed with RRL. **(D)** 102-mer pOTC RNCs with BpA at positon H18 were synthesized in RRL and separated from bulk cytosol under high-salt conditions via centrifugation. The resuspended RNCs were mixed with or without cytosolic extract from ΔST13 HEK cells and subjected to UV irradiation. Samples were then subjected to IP with antibodies against the substrate (FLAG) or Hsc70.

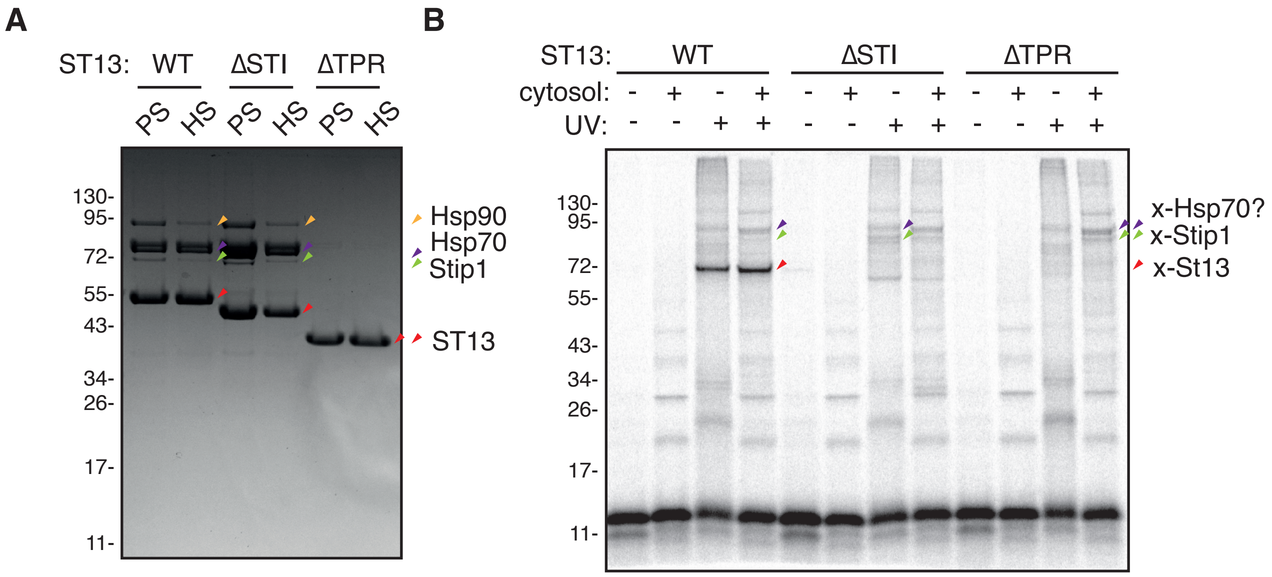

Figure S5. **St13 engages MTS via its STI1 domain.**
(A) FLAG-tagged ST13 recombinant proteins (WT, ΔSTI1, ΔTPR) were produced in HEK cells and purified under physiological salt (PS) or high salt (HS) conditions. Purified proteins were analyzed by SDS-PAGE and Coomassie staining. ST13 mutants are marked with red arrows, whereas known interacting partners are labeled as follows: Hsc70 is marked with purple arrows, Stip1 with green arrows and Hsp90 with orange arrows. Note the disapperance of interacting partners in the ΔTPR mutant. **(B)** Ribosome-nascent chain complexes (RNCs) of 102-mer pOTC were produced in RRL and purified by centrifugation through a sucrose cushion in high salt buffer. Stripped RNCs were then resuspended in buffer containing the indicated purified proteins from the high salt condition in (A) alone or together with cytosolic extracts from ΔST13 cells. Note the strongest signal for St13 crosslink when WT protein was added (even in the absence of the cytosol).
